# Central angiotensin 1–7 triggers brown fat thermogenesis

**DOI:** 10.14814/phy2.15621

**Published:** 2023-03-10

**Authors:** F. S. Evangelista, T. J. Bartness

**Affiliations:** ^1^ School of Arts, Science and Humanities University of Sao Paulo Sao Paulo Brazil; ^2^ Department of Biology, Center for Obesity Reversal, Neuroscience Institute Georgia State University Atlanta Georgia USA

**Keywords:** brown adipose tissue, central angiotensin 1–7, lipolysis and thermogenesis, Mas receptor

## Abstract

We tested the hypothesis that third ventricular (3V) injections of angiotensin 1–7 (Ang 1–7) increases thermogenesis in brown adipose tissue (BAT), and whether the Mas receptor mediates this response. First, in male Siberian hamsters (*n* = 18), we evaluated the effect of Ang 1–7 in the interscapular BAT (IBAT) temperature and, using selective Mas receptor antagonist A‐779, the role of Mas receptor in this response. Each animal received 3V injections (200 nL), with 48 h intervals: saline; Ang 1–7 (0.03, 0.3, 3, and 30 nmol); A‐779 (3 nmol); and Ang 1–7 (0.3 nmol) + A‐779 (3 nmol). IBAT temperature increased after 0.3 nmol Ang 1–7 compared with Ang 1–7 + A‐779 at 20, 30, and 60 min. Also, 0.3 nmol Ang 1–7 increased IBAT temperature at 10 and 20 min, and decreased at 60 min compared with pretreatment. IBAT temperature decreased after A‐779 at 60 min and after Ang 1–7 + A‐779 at 30 and 60 min compared with the respective pretreatment. A‐779 and Ang 1–7 + A‐779 decreased core temperature at 60 min compared with 10 min. Then, we evaluated blood and tissue Ang 1–7 levels, and the expression of hormone‐sensitive lipase (HSL) and adipose triglyceride lipase (ATGL) in IBAT. Male Siberian hamsters (*n* = 36) were killed 10 min after one of the injections. No changes were observed in blood glucose, serum and IBAT Ang 1–7 levels, and ATGL. Ang 1–7 (0.3 nmol) increased p‐HSL expression compared with A‐779 and increased p‐HSL/HSL ration compared with other injections. Ang 1–7 and Mas receptor immunoreactive cells were found in brain regions that coincide with the sympathetic nerves outflow to BAT. In conclusion, 3V injection of Ang 1–7 induced thermogenesis in IBAT in a Mas receptor‐dependent manner.

## Graphical abstract

Central injection of Ang 1‐7 induces thermogenesis in the brown adipose tissue in a Mas receptor‐dependent manner. Our data contribute to the investigation of new therapies for obesity.

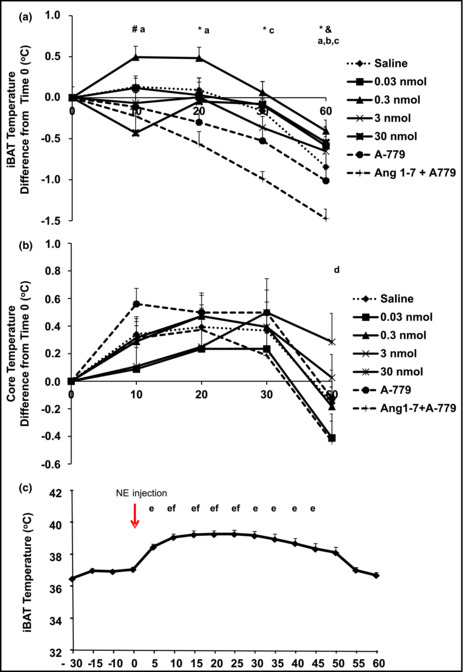

## 1 INTRODUCTION

Effective strategies to treat obesity and its comorbidities are a critical point facing medical science. The stimulation of metabolically active brown adipose tissue (BAT) or the increase of brown‐like adipocytes (browning) can improve energy expenditure, decrease adiposity, and ameliorate metabolic complications of obesity and Type 2 Diabetes Mellitus (T2DM; Gaspar et al., 2021; Yoneshiro et al., 2013), since BAT is a tissue that consumes amounts of fatty acids and glucose as fuel for thermogenesis. In fact, in obese individuals, both increased BAT activation and browning could improve lipid oxidation to generate heat and prevent lipid accumulation (Scheel et al., 2022). Additionally, by increasing peripheral glucose uptake, the BAT activation has become a potential target for improving insulin sensitivity in patients with T2DM (Hanssen et al., 2015).

The renin–angiotensin system (RAS) has emerged as an important target to treat obesity (Morimoto et al., 2018; Oliveira Andrade et al., 2014). The components of the RAS represented by angiotensin converting enzyme 2 (ACE2)/angiotensin 1–7 (Ang 1–7)/ Mas receptor axis expanded the role of the RAS in many physiological and pathophysiological processes. ACE2 can cleave Ang 1 to generate the inactive Ang 1–9 peptide, which then can be converted to the peptide Ang 1–7 by ACE or other peptidases. ACE2 also can directly generate Ang 1–7 from Ang 2. By binding to its specific Mas receptor, Ang 1–7 can cause vasodilation, and antiproliferative, antihypertrophic, antifibrotic, and antithrombotic effects (Santos et al., 2003, 2018; Xia & Lazartigues, 2008).

Ang 1–7 treatment has been shown to enlarge BAT and increase the expression of UCP1, PRDM16, and prohibitin. Also, Ang 1–7 treatment induces brown adipocyte differentiation leading to upregulation of thermogenesis and better metabolic profile in high‐fat‐fed mice (Morimoto et al., 2018). Recently, Cao et al. (2022) demonstrated that Ang 1–7‐treated Lepr^db/db^ and the high‐fat‐fed‐induced obese mice were better able to regulate their body temperature during cold stress and had increased UCP1 expression in the BAT compared to the control. They also showed that the increase in UCP1 brown adipocytes was induced by ACE2 pathway activated Akt/FoxO1 and PKA pathway (Cao et al., 2022).

Although the potential therapeutic properties of Ang 1–7 for the treatment of obesity have been elucidated, a knowledge gap still exists about the effect of central Ang 1–7 on adipose tissue metabolism. The first study involving Ang 1–7 peptide in the central nervous system was developed from Fitzsimons (1971), which showed that Ang 1–7 has no dipsogenic effect when injected into the rat brain. Subsequent studies showed the role of Ang 1–7 in the hypothalamic paraventricular nucleus (PVH) and its interaction with neurons that play a pivotal role in cardiovascular regulation (Ambuhl et al., 1994; Han et al., 2012; Silva et al., 2005).

Considering that BAT is innervated by the sympathetic nervous system (Bamshad et al., 1999), the sympathetic neuronal release of norepinephrine activates thermogenesis in the BAT (Contreras et al., 2014), and the potential of central Ang 1–7 to activate sympathetic neurons (Ambuhl et al., 1994; Fitzsimons, 1971; Han et al., 2012; Silva et al., 2005), we hypothesized that central Ang 1–7 modulates the metabolic activity of BAT. Thus, the aim of this study was to test whether third ventricular (3V) injections of Ang 1–7 increases BAT thermogenesis, and whether the Mas receptor mediates this response.

## 2 MATERIALS AND METHODS

### 2.1 Animals

Fifty‐four (*n* = 54) adult male Siberian hamsters (*Phodopus sungorus*), 3–4 months old, were obtained from the breeding colony of the laboratory of Prof. Timothy Bartness. The hamsters were single housed in plastic, and maintained under a long‐day photoperiod (16‐h light, 8‐h dark, and lights on at 3:00 h) at 22 ± 1.5°C. Food (Purina Rodent Chow no. 5001) and tap water were available ad libitum throughout the experiment. Housing and all procedures were approved by the Georgia State University Institutional Animals Care and Use Committee (protocol #A12056), and were in accordance with the Public Health Service and U.S. Department of Agriculture guidelines.

### 2.2 Intraventricular cannula implantation

Cannulae were stereotaxically implanted into the 3V, as described previously (Day & Bartness, 2004). Briefly, the animals were anesthetized with isoflurane 2%, and the fur at the top of the head was removed to expose the area to be incised. After exposure of the skull, a hole was trephined at the intersection of bregma and the midsagittal sinus and the guide cannula (26 gauge stainless steel; Plastics One) was positioned using the following stereotaxic coordinates: level skull, anterior‐lateral from bregma, 0 mm; medial‐lateral from midsagittal sinus, 0 mm; and dorsal–ventral, −5.5 mm from the top of the skull, which targeted placement just above the 3V. The guide cannula was secured to the skull with 3/16 mm jeweler's screws, cyanoacrylate glue, and dental acrylic. A removable obturator (Plastics One) sealed the opening in the guide cannula throughout the experiment, except when it was removed for the injections. After the surgeries, the hamsters were transferred to clean biohazard cages and received subcutaneous injections of ketofen (5 mg/kg; Fort Dodge Animal Health), an analgesic, for 3 days. They also received apple slices to supply readily consumed calories and water.

### 2.3 Temperature transponder and iButton implants

In a group of hamsters (*n* = 18), temperature transponder and iButton were also implanted at the same time as the cannula implantation. These animals were assigned to the procedures of Experiment 1.

The temperature transponder (Implantable Programmable Temperature Transponder 300 [IPTT‐300]; BioMedic Data Systems) was implanted under the IBAT, such that temperature from both pads could be measured. For this, the fur around the scapula was shaved, and the skin was wiped with povidone iodine (Ricca Chemical) and alcohol, and then again with povidone iodine. The hamsters were placed in ventral recumbency, and a subcutaneous incision was made to reveal IBAT. The IBAT was gently pulled out onto an isotonic saline‐soaked sterile surgical drape to prevent desiccation, and the transponder was positioned under the IBAT, and secured to the surrounding muscle with sterile suture (Ethicon, Johnson & Johnson). The skin was closed with sterile wound clips (Stoelting), and nitrofurazone powder (nfz Puffer, Hess and Clark) was applied to minimize infection. Then, the animal was shaved on the ventral side, and placed supine. A vertical incision was made through the skin and the peritoneum to expose the abdominal cavity. The temperature probe iButton was introduced in the abdominal cavity and positioned above the intestines. The peritoneum and skin were closed with sterile sutures and sterile wound clips, respectively. Nitrofurazone powder was applied to minimize infection. The iButton was programmed to monitor the abdominal temperature every 10 min, automatically and continuously. Animals were excluded from the analysis if the temperature transponder or iButton were out of position at the time of death.

### 2.4 Experiment 1: Injection protocol and temperature measurements

Experiment 1 aimed to determine the IBAT temperature response to doses of Ang 1–7 and, by the use of selective Mas receptor antagonist A‐779, the role of the Mas receptor in changes of IBAT temperature induced by Ang 1–7. Two weeks postcannulation, the animals were adapted to the handling procedure for the ICV injections, once each day, for 10 days. In addition, the temperature sensing wand also was used to adapt the animals to several low beeping sounds produced by the recording apparatus (DAS 5002 Notebook System; BioMedic Data Systems), when acquiring the IBAT temperature.

On the test day at 0700 h, food was removed from the pouches of the hamsters and from their cages, but water was present. Two hours later, the temperature of IBAT was measured to determine the beginning baseline (time 0). After that, hamsters received immediately a single injection of either vehicle (sterile 0.15 M NaCl) or one of the four doses (0.03, 0.3, 3.0, or 30 nmol) of Ang 1–7 (Bachem), or 3 nmol of (D‐Ala^7^)‐Angiotensin I/II (1–7; A‐779) selective Mas receptor antagonist (Bachem), or Ang 1–7 (0.3 nmol) + A‐779 (3 nmol). The order of the vehicle and drug injections was counterbalanced to minimize drug order effects. The injection volume for the vehicle (sterile saline) or drugs was 200 nL, and each animal received all injections with an interval of 48 h between injections to minimize carryover effects. IBAT temperature was assessed at 10, 20, 30, and 60 min by passing the temperature sensing wand 10–20 mm above the back of the animal in its cage. At the end of the series of injections, an additional 48 h washout period occurred before each hamster was injected peripherally with 0.8 mg/kg of the β‐adrenoceptor agonist, norepinephrine, and IBAT temperature recorded for 60 min as a positive control to assess transponder function.

After the end of the last test, the hamsters were killed by overdose with an intraperitoneal injection of pentobarbital sodium (300 mg/kg), and transcardial perfusion was performed with heparinized 0.02% saline (75 mL), followed by 4% paraformaldehyde (150 mL) solution. Evans blue dye (200 nL) was injected into the cannulae to confirm placement of the cannula in the 3V. The brains were removed and placed in the same fixative overnight, and then immersed in a cryoprotectant solution of 30% sucrose. Each brain was sliced at 40 μm in a freezing stage sliding microtome, then stained with cresyl violet. Animals were included in the analysis if the dye was visible in any part of the 3V.

### 2.5 Experiment 2: Injection protocol and metabolic measurements

Another set of hamsters (*n* = 36) was used to evaluate blood and tissue concentration of Ang 1–7, and the expression of hormone‐sensitive lipase (HSL) and adipose triglyceride lipase (ATGL), which are markers of lipolysis due to their role in triacylglycerol hydrolysis. All fitting procedures followed as described above, except for the absence of temperature transponder and iButton implant. On the test day at 0700 h, food was removed from the pouches of the hamsters and from their cages, but water was present. The hamsters were weighted and 2 h later, they received one single injection (200 nL) of vehicle (sterile 0.15 M NaCl), 0.3 nmol of Ang 1–7 (dose determined from its effect on IBAT temperature in the Experiment 1), and 3 nmol of A‐779 or Ang 1–7 (0.3 nmol) + A‐779 (3 nmol). The hamsters were decapitated 10 min postinjection (time determined from the IBAT temperature response in the Experiment 1), and trunk blood was collected and stored on ice. Blood glucose was measured using a glucometer (AccuChek Advantage Roche Diagnostics®). IBAT was excised, immediately minced on dry ice, snap frozen in dry ice, and stored at −80°C until analysis. Immediately after decapitation, 200 nL of Evans blue dye was injected into the cannulae to confirm placement of the cannula in the 3V. The brains were removed and postfixed in 4% paraformaldehyde for a minimum of 1 week before the slice procedure. Each brain was sliced at 40 μm in a freezing stage sliding microtome, then stained with cresyl violet. Animals were included in the analysis if the dye was visible in any part of the 3V.

### 2.6 Ang 1–7 dosage

The concentration of Ang 1–7 was measured in serum and IBAT using a commercial peptide enzyme immunoassay kit (MyBioSource Inc), following the manufacturer's instructions (Dilauro et al., 2010).

### 2.7 Western blotting

IBAT was homogenized in lipid‐associated protein extraction buffer according to (Sherestha et al., 2010) containing 50 mM HEPES, 100 mM NaCl, 10% SDS, 2 mM EDTA, 0.5 mM DTT, 1 mM benzamidine, protease inhibitor cocktail (Calbiochem, EMD Chemicals) at 50 μL/g of tissue, and phosphatase inhibitor cocktail (Halt; Pierce, Thermo Fisher Scientific, Rockford, IL). After the incubation and centrifugation procedures, the infranatant containing the protein extract was aliquoted, and stored at −80°C. Protein content was determined by bicinchoninic acid protein assay kit (Thermo Fisher Scientific). Samples were loaded and subjected to electrophoresis, then were transferred to the membrane. The blot membrane was incubated with 4% nonfat dry milk in Tris‐buffered saline, for 2 h, at 4°C, and then incubated overnight, at 4°C, with anti‐phospho‐HSL (Ser660; Cell Signaling Technology, Boston, MA, USA), anti‐HSL (Cell Signaling Technology), anti‐ATGL (Cell Signaling Technology), and anti‐β‐actin (Cell Signaling Technology). Binding of the primary antibody was detected with the use of anti‐rabbit IgG HRP‐linked secondary antibody (Cell Signaling Technology), at 4°C, for 2 h. The antibodies were diluted at 1:1000. Finally, the membranes were washed in TTBS for 3 × 10 min, and incubated with the chemiluminescent Lumiglo Reagent (Cell Signaling Technology), for 5 min. The bands on the membrane were visualized using an Image Quant LAS 4000 mini system (GE Healthcare Life Sciences®). Band intensities were quantified based on optical densitometry measurements using the ImageJ program (version 1.43 for Windows).

### 2.8 Ang 1–7 and Mas receptor immunostaining

This procedure was done in four hamsters (*n* = 4) to evaluate the anatomical localization of Ang 1–7 and Mas receptor in the brain. The animals were killed and transcardially perfused with heparinized 0.02% saline (100 mL), followed by 4% paraformaldehyde solution pH 7.4 (150 mL). The brains were removed and postfixed in 4% paraformaldehyde for 3 h, and then placed in 30% sucrose solution. Coronal sections (30 μm) were prepared using a freezing stage sliding microtome.

Free‐floating brain sections were rinsed in 0.1 M PBS (2 × 15 min) followed by a 10 min incubation in 10% MeOH, 0.3% H_2_O_2_, and 0.1 M PBS. Next, sections were rinsed again in 0.1 M PBS (2 × 15 min), followed by a 1 h incubation in 2% normal goat serum (NGS) in 0.1 M PBS. Sequentially, sections were incubated with primary antibody for rabbit anti‐Ang 1–7 (1:500; Phoenix Pharmaceuticals Inc.) or anti‐Mas receptor (1:200; Alomone Labs), with 0.3% Triton X‐100, 2% NGS in 0.1 M PBS at 4^0^ for 16 h. Next, slices were rinsed in 0.1 M PBS (2 × 15 min), incubated for 1 h in 2‐biotinylated goat anti‐rabbit secondary antibody (1:200; Vector Laboratories) in 2% NGS in PBTx, rinsed in 0.1 M PBS (2 × 15 min), and incubated for 1 h in avidin and biotin (1:800) in 0.1 M PBS. Thereafter, the sections were rinsed in 0.1 M phosphate buffer (PB; 2 × 15 min), following incubation with 3,3′ diaminobenzidine (DAB) in 0.1 M PB, during 5 min. Finally, the sections were rinsed in 0.1 M PB (2 × 15 min), mounted onto slides, and cover slipped using Permount.

### 2.9 Statistical analyses

Data were reported as mean ± SE. The results of IBAT were compared using two‐way analyses of variance (ANOVA) for repeated measures. The other variables were compared using one‐way ANOVA. The Tukey post hoc test was used to determine differences among means when a significant change was observed with ANOVA. A *p* ≥ 0.05 was statistically significant.

## 3 RESULTS

**FIGURE 1:**
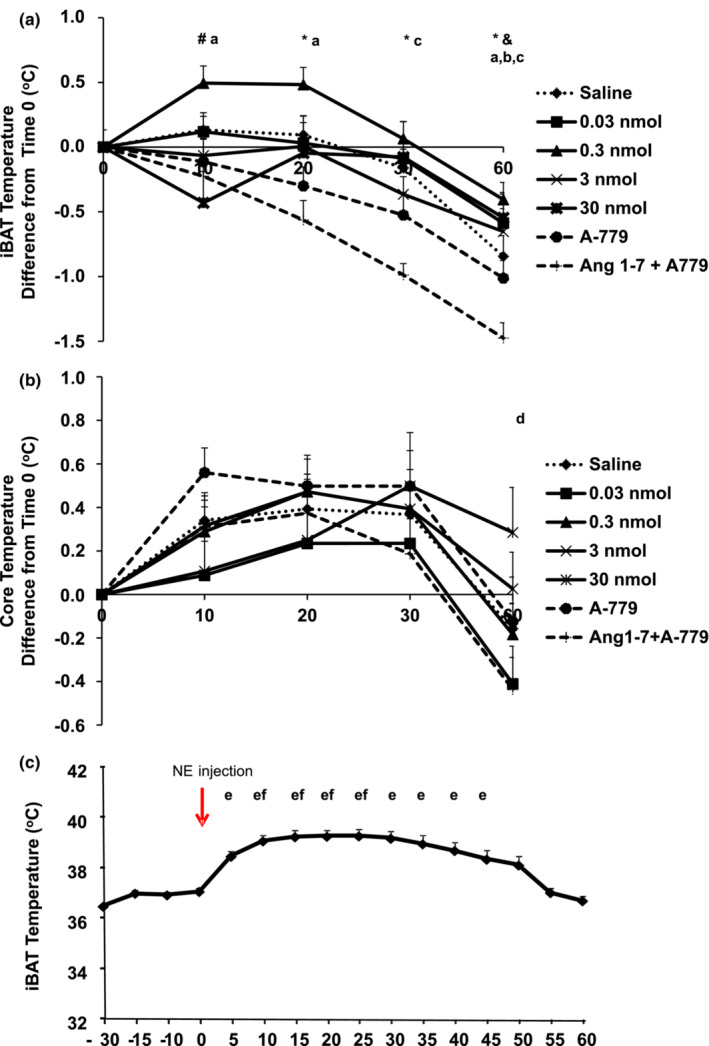
Difference in IBAT temperature (a) and core temperature (b) before (time 0) and after 3V injections; IBAT temperature after norepinephrine injection (c).Error bars indicate the SE. Saline (*n* = 18), Ang 1–7 0.03 nmol (*n* = 16), 0.3 nmol (*n* = 17), 3 nmol (*n* = 16), 30 nmol (*n* = 13), A‐779 (*n* = 8), and Ang 1–7 + A‐779 (*n* = 8). *p ≤ 0.05 0.3 nmol Ang 1–7 versus Ang 1–7 + A‐779 at 20′, 30′ and 60′; ^#^ *p* ≤ 0.05 0.3 nmol Ang 1–7 versus 30 nmol Ang 1–7 at 10′; ^&^ *p* ≤ 0.05 30 nmol Ang 1–7 versus Ang 1–7 + A‐779 at 60′; ^a^ *p* ≤0.05 0.3 nmol Ang 1–7 at 0′ versus 10′, 20′ and 60′; ^b^ *p* ≤ 0.05 A‐779 at 0′ versus 60′; ^c^ *p* ≤ 0.05 Ang 1–7 + A‐779 at 0′ versus 30′ and 60′; ^d^ *p* ≤0.05 A‐779 and Ang 1–7 + A‐779 60′ versus 10′; ^e^ *p* ≤ 0.05 versus 0′; ^f^ *p* ≤ 0.05 versus 5′. IBAT , interscapular brown adipose tissue; 3V = third ventricular. IBAT and core temperature were compared by two‐way ANOVA and IBAT temperature after norepinephrine was compared by one‐way ANOVA. The Tukey post hoc test was used to determine differences among values.

The results of Experiment 1 are shown in the Figure 1. Comparisons among different treatments were performed at the same time point, and IBAT temperature was higher after 0.3 nmol Ang 1–7 than Ang 1–7 + A‐779 at 20, 30, and 60 min. The 0.3 nmol Ang 1–7 injection also significantly increased IBAT temperature compared with 30 nmol Ang 1–7 at 10 min, and 30 nmol Ang 1–7 injection significantly increased IBAT temperature compared with Ang 1–7 + A‐779 at 60 min (Figure 1a). Comparisons in each time point of the treatment versus immediately before the same treatment (pretreatment) revealed that IBAT temperature from animals injected with 0.3 nmol Ang 1–7 significantly increased at 10 min and 20 min compared with pretreatment but decreased at 60 min compared with pretreatment. The injection of A‐779 decreased IBAT temperature at 60 min compared with pretreatment, while the injection of Ang 1–7 + A‐779 decreased IBAT temperature at 30 min and 60 min compared with pretreatment (Figure 1a). Third ventricular A‐779 and Ang 1–7 + A‐779 injections significantly decreased abdominal temperature at 60 min compared with 10 min (Figure 1b). Intra‐treatment differences were not observed in abdominal temperature (Figure 1b). The test with norepinephrine revealed proper temperature transponder functioning because IBAT temperature significantly increased at 5, 10, 15, 20, 25, 30, 35, 40, and 45 min after norepinephrine injection (Figure 1c).

No differences in body weight and fat pads weight were found among animals in the Experiment 2 (Table 1). Blood glucose concentration did not change after 10 min of all 3V injections (Table 2). Also, the concentration of Ang 1–7 in the serum and BAT did not change in the animals after 3V injections (Table 2).

**TABLE 1:** Body weight and the weight of fat pads evaluated at the time of death of the animals injected with sterile saline, Ang 1–7, A‐779, or Ang 1–7 + A‐779.

	Saline (*n* = 10)	Ang 1–7 (*n* = 8)	A‐779 (*n* = 9)	Ang 1–7 + A‐779 (*n* = 9)
Body weight (g)	42 ± 1.72	42 ± 2.37	43.6 ± 1.83	44 ± 1.33
IBAT (g)	0.17 ± 0.02	0.14 ± 0.02	0.16 ± 0.02	0.18 ± 0.02
IWAT (g)	1.29 ± 0.11	1.25 ± 0.22	1.27 ± 0.19	1.32 ± 0.21
RWAT (g)	0.13 ± 0.02	0.14 ± 0.02	0.13 ± 0.02	0.12 ± 0.02
EWAT (g)	0.52 ± 0.09	0.70 ± 0.15	0.43 ± 0.06	0.50 ± 0.13

*Note*: Data are presented as mean ± SE. The results were compared by one‐way ANOVA plus Tukey post hoc test.

Abbreviations: EWAT, epididymal white adipose tissue; IBAT, interscapular brown adipose tissue; IWAT, inguinal white adipose tissue; RWAT, retroperitoneal white adipose tissue.

**TABLE 2:** Blood glucose and Ang 1–7 concentration in serum and IBAT after 3V injections of sterile saline, Ang 1–7, A‐779, or Ang 1–7 + A‐779. Animals were killed 10 min after receiving one of the injections.

	Saline (*n* = 4–10)	Ang 1–7 (*n* = 6–8)	A‐779 (*n* = 4–9)	Ang 1–7 + A‐779 (*n* = 4–9)
Blood glucose (mg/dL)	149 ± 9.2	155 ± 16.9	146 ± 17.1	146 ± 8.1
Blood Ang 1–7 (ng/mL)	3.6 ± 1.9	5.1 ± 1.1	2.2 ± 0.6	2.1 ± 1.4
IBAT Ang 1–7 (ng/mg)	3.2 ± 0.7	3.0 ± 1.2	4.4 ± 0.7	3.3 ± 1.0

*Note*: Data are presented as mean ± SE. The results were compared by one‐way ANOVA plus Tukey post hoc test.

Abbreviations: IBAT, interscapular brown adipose tissue; 3V, third ventricular.

**FIGURE 2:**
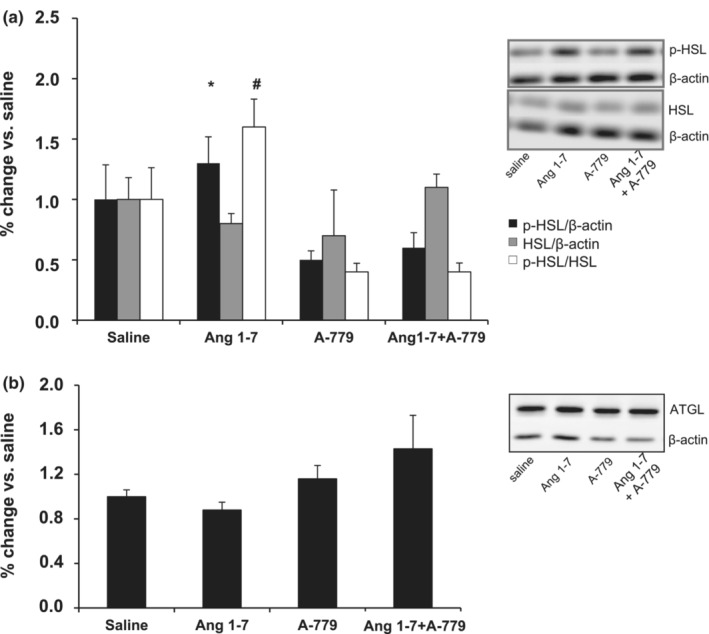
p‐HSL, total HSL, p‐HSL/HSL (a), and ATGL (b) expression in the IBAT.Immunoblotting data are shown as the percentage change compared with saline (*n* = 4–7). Error bars indicate the SE. *p ≤ 0.05 versus A‐779; ^#^ *p* ≤ 0.05 versus saline, A‐779, and Ang 1–7 + A‐779. HSL = hormone‐sensitive lipase; ATGL = adipose triglyceride lipase. The results were compared by one‐way ANOVA plus Tukey post hoc test.

As shown in Figure 2a, the p‐HSL expression increased in IBAT after Ang 1–7 injection compared with A‐779. We also found a significant increase in the ratio of p‐HSL to total HSL in IBAT after Ang 1–7 injection compared with saline, A‐779, and Ang 1–7 + A‐779. Injection of Mas receptor antagonist A‐779 + Ang 1–7 blocked the effect of Ang 1–7 on the expression of p‐HSL to total HSL ratio (Figure 2a). The level of ATGL expression in IBAT did not modify after injections of Ang 1–7, A‐779, and Ang 1–7 + A‐779 (Figure 2b).

**FIGURE 3:**
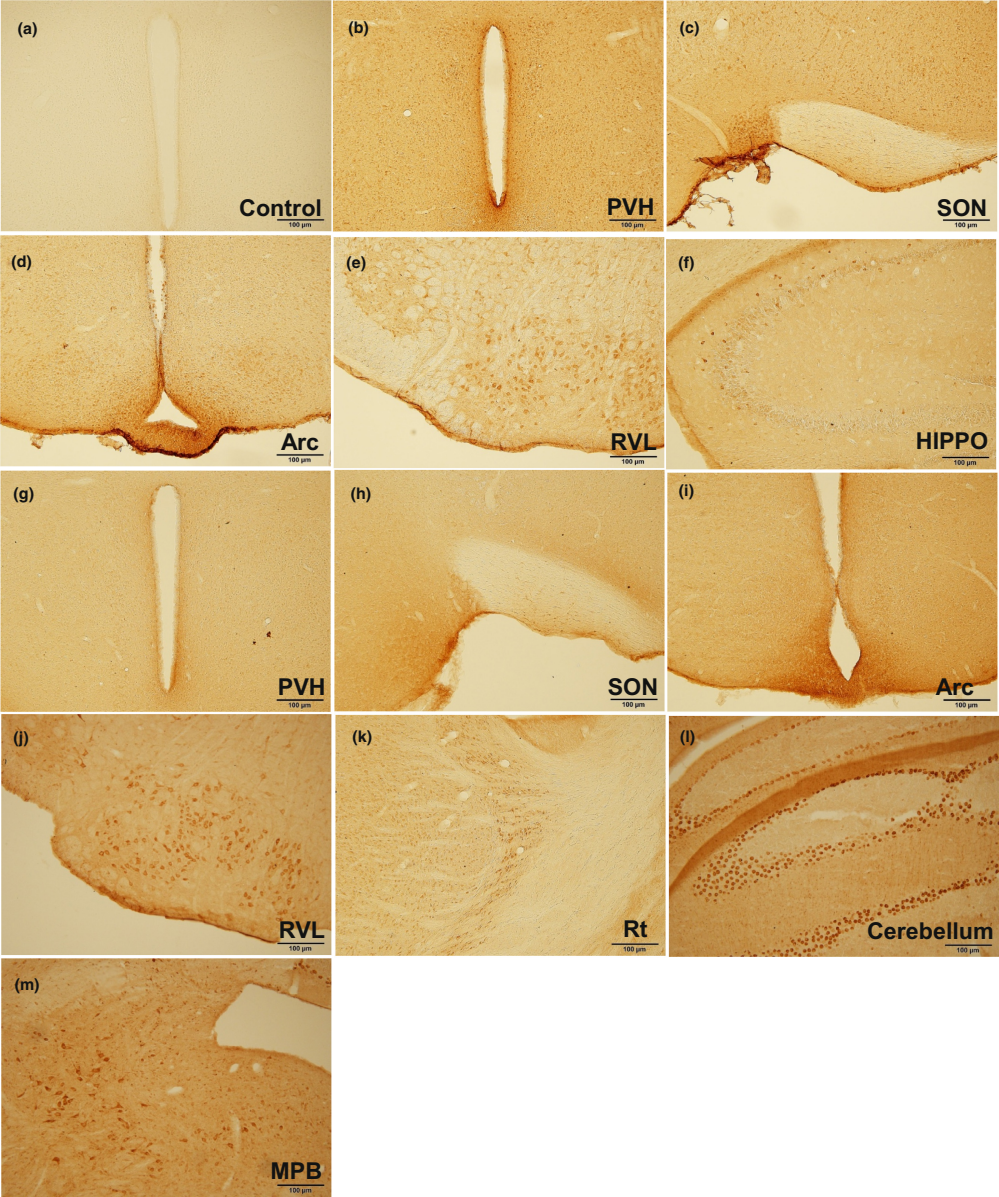
Ang 1–7 (b–e) and Mas receptor (f–m) immunostaining in the brain. Control section was incubated without primary antibodies (a). Arc, arcuate hypothalamic nucleus; HIPPO, hippocampus; MPB, medial parabrachial nucleus. PVH, hypothalamic paraventricular nucleus; Rt, reticular nucleus; RVL, rostro ventrolateral reticular nucleus; SON, supraoptic nucleus;

The distribution of Ang 1–7 immunoreactive cells is shown on representative sections through the forebrain in Figure 3b–e. Labeled cells were observed in the hypothalamic paraventricular nucleus (PVH), supraoptic nucleus (SON), arcuate hypothalamic nucleus (Arc), and rostroventral lateral reticular nucleus (RVL). Omission of the primary antibody did not reveal any specific staining (negative control; Figure 3a). In addition, Mas receptor immunoreactive cells were found in different areas of the Siberian hamster brain, including hippocampus (HIPPO), PVH, SON, Arc, RVL, reticular nucleus (Rt), cerebellum, and medial parabrachial nucleus (MPB; Figure 3f–m).

## 4 DISCUSSION

In this research, we investigated whether 3V injection of Ang 1–7 could increase IBAT temperature in a conscious hamster model. Our results showed that 0.3 nmol Ang 1–7 significantly increased IBAT temperature at 10 and 20 min after injection. In addition, the blockade of Mas receptor with A‐779 did not change IBAT temperature in the first 20 min, but the effect of 0.3 nmol Ang 1–7 was lost when the 3V injection was combined with A‐779, confirming the action of Ang 1–7 in a Mas receptor‐dependent manner.

In a previous study, Silva et al. (2005) showed that injection of A‐779 into the PVN reduced sympathetic activity and that the pressor effect produced by Ang 1–7 was blocked by A‐779. These results indicated that the actions of endogenous Ang 1–7 are mediated by the Mas receptor, since A‐779 is a selective Mas receptor antagonist. In addition, Santos et al. (2018) described that Ang 1–7 actions in the brain are mainly mediated by its interaction with the Mas receptor. In line with previous studies, our results reinforce the role of the Mas receptor in mediating the central actions of Ang 1–7.

The effect of central Ang 1–7 seems to be short according to our study and the literature. Silva et al. (2005) found significant increase in the renal sympathetic nerve activity after 30 min of Ang 1–7 injection in the PVH. The same effect was observed by Han et al. (2012) between 7 and 12 min. It is important to note that the Ang 1–7 concentration used in our study was less than the concentration used by Han et al. (2012), which observed bigger response in renal sympathetic nerve activity and blood pressure after PVH injection of 3 nmol Ang 1–7 compared with 0.03 nmol and 0.3 nmol.

IBAT temperature started to drop after 30 min with some significant differences in 0.03 nmol Ang 1–7, 3 nmol Ang 1–7, A‐779, and Ang 1–7 + A‐779 compared with the pretreatment values (time 0). Both A‐779 and Ang 1–7 + A‐779 injections significantly decreased core temperature at 60 min compared with 10 min, which can be associated with IBAT temperature reduction in these groups. The drop in IBAT temperature in response to A‐779 alone is likely due to the antagonist having an opposing action on IBAT relative to the agonist and the blockade of endogenous Ang 1–7, since the endogenous Ang 1–7 acting in the PVN neurons may contribute to the maintenance of sympathetic activity, as showed by Silva et al. (2005). Other factors can explain the lower core temperature such as reductions in stress and movement. In fact, some of the animals slept at the end of the measurement period. Although they had an adaptation period before the temperature tests, handling the animals during injection can cause a minimum stress reflecting in the temperature. Interestingly, we did not observe a decrease in IBAT temperature after 0.3 nmol Ang 1–7 injection.

According to the IBAT temperature responses, we determined the concentration of 0.3 nmol of Ang 1–7 to develop the second set of experiment in animals, separated in groups matched for body weight and fat mass.

Peripheral Ang 1–7 has been associated with increases in glucose uptake in insulin‐target tissues and insulin resistance prevention (Cao et al., 2014; Liu et al., 2011; Munoz et al., 2012). Our data demonstrated that 3V injection of Ang 1–7 did not change blood glucose concentration. If the central Ang 1–7 is indeed increasing the sympathetic drive, a possible explanation for unchanged glycemia is the augment in liver glycogenolysis, which counterbalances the reduction in blood glucose concentration induced by BAT sympathetic activation (Chaves et al., 2006; La Fleur et al., 2000). Considering that sympathetic nerve activity was not evaluated in this study, further experiments are needed to confirm this response.

Lipolysis provides substrates that are required for BAT thermogenesis (Labbé et al., 2015). The phosphorylated HSL (p‐HSL) is a potential intracellular marker of catecholamine‐stimulated lipolysis due to the critical role in triacylglycerol hydrolysis (Sherestha et al., 2010). Specifically, catecholamines induce lipolysis in adipocytes after binding to adrenoceptor subtypes beta‐1, beta‐2, and beta‐3 (in rodents, primarily beta 3; Lonnqvist et al., 1995), which are linked to G proteins. Beta‐adrenoceptors are coupled to stimulatory G proteins and activate adenylate cyclase, increasing the production of cyclic adenosine monophosphate (cAMP), and thus activating the protein kinase A (PKA). PKA phosphorylates HSL and perilipin, a protein on the surface of lipid droplets in adipocytes, allow the hydrolysis of TAG (Arner, 2005; Duncan et al., 2007). In addition, PKA‐induced p‐HSL also is necessary for the BAT thermogenesis induced by sympathetic activity (Souza et al., 2007). Therefore, it seems that levels of p‐HSL could serve as fat pad‐specific in vivo indicator of catecholamine‐induced BAT thermogenesis (Sherestha et al., 2010). We showed that central Ang 1–7 increased p‐HSL in IBAT compared with A‐779 and increased the ratio of p‐HSL to total HSL in IBAT compared with saline, A‐779, and Ang 1–7 + A‐779. These results allowed us to speculate that central Ang 1–7 can control BAT thermogenesis through the sympathetic nervous activity in a Mas receptor‐dependent manner. However, new studies are still necessary to demonstrate the Mas receptor expression in sympathetic neurons that project to BAT.

Other proteins are responsible for lipolysis together with HSL, such as ATGL and monoacylglycerol lipase (MAGL). ATGL initiates lipolysis by breaking the first fatty acid from TAG, and then HSL and MAGL act on diacylglycerol and monoacylglycerol, respectively (Lafontan & Langin, 2009). ATGL is predominantly involved in basal lipolysis and appears to be required for all PKA‐stimulated free fatty acid release in the absence of HSL. Although ATGL seems important for cAMP‐dependent PKA stimulation of free fatty acid release, without the activation of HSL and perilipin A, the complete lipolysis of triacylglycerol to its hydrolytic end products of glycerol and free fatty acid cannot be achieved. We found no changes in the level of ATGL expression in IBAT after injections of Ang 1–7, A‐779 and Ang 1–7 + A‐779. This result corroborates the study by (Morimoto et al., 2018), in which it was shown that Ang 1–7 decreased lipid droplet size in the BAT concomitant with increased HSL phosphorylation, but without altering ATGL levels.

To advance knowledge about the components of RAS in the central nervous system, we performed an exploratory analysis to identify the distribution of Ang 1–7 and Mas receptor immunoreactive cells in the brain of the Siberian hamster. According to our data, both Ang 1–7 and Mas receptor cells were found in regions that coincide with the localization of sympathetic nerves that innervate both white and brown adipose tissue. In fact, previous studies showed connected neurons extending from the forebrain to the fat pads, including several hypothalamic, midbrain, and brain stem regions, as well as the spinal cord. Labeled neurons were found in the paraventricular nucleus, suprachiasmatic nucleus, medial preoptic area, dorsomedial hypothalamic nucleus, ventromedial hypothalamic nucleus, and arcuate hypothalamic nucleus (Bamshad et al., 1998, 1999). They also found cells in areas of the brain stem such as the nucleus of the solitary tract, raphe obscurus nucleus, lateral reticular nucleus and rostro ventrolateral reticular nucleus, and in the midbrain (central gray).

Although we have not evaluated the injections sites, the coexpression of Mas receptor with neurons that are part of the sympathetic outflow from brain to BAT, and the ability of Mas receptor agonism in these sites to stimulate lipolysis and thermogenesis can open perspectives for new discoveries about the connection between Ang 1–7 and sympathetic nervous system in the control of BAT metabolism. Our and other immunochemistry results demonstrated this connection.

## 5 CONCLUSION

In conclusion, 3V injection of Ang 1–7 induced thermogenesis in BAT in a Mas receptor‐dependent manner. These data provide strong evidence of central Ang 1–7 for BAT thermoregulation and contribute to the investigation of new therapies for obesity.

## CONFLICT OF INTEREST STATEMENT

The authors declare that there are no conflicts of interest.

## ETHICAL STATEMENT

Studies involving animals in this article were approved by the Georgia State University Institucional Animals Care and Use Committee.
